# Rationale and design of the STOP-IMH randomised trial: Safety of ticagrelor monotherapy after primary percutaneous coronary intervention for ST-elevation myocardial infarction and the effect on intramyocardial haemorrhage

**DOI:** 10.1016/j.ijcha.2024.101564

**Published:** 2024-11-22

**Authors:** E.C.I. Woelders, B. Yosofi, D.A.M. Peeters, L.S.F. Konijnenberg, C. von Birgelen, J.B. van Rees, S.C.H. van den Oord, A.A.C.M. Heestermans, B.E.P.M. Claessen, N. van Royen, R.J.M. van Geuns, R. Nijveldt, P. Damman

**Affiliations:** a Radboud University Medical Centre, Department of Cardiology, Nijmegen, The Netherlands; b Medisch Spectrum Twente, Department of Cardiology, University of Twente, Health Technology and Services Research, Faculty BMS, Enschede, The Netherlands; c Rijnstate Ziekenhuis, Department of Cardiology, Arnhem, The Netherlands; d Noordwest Ziekenhuisgroep, Department of Cardiology, Alkmaar, The Netherlands; e Amsterdam UMC, locatie AMC, Department of Cardiology, Amsterdam, The Netherlands

**Keywords:** STEMI, Ticagrelor monotherapy, Intramyocardial haemorrhage, Cardiac magnetic resonance imaging

## Abstract

**Background:**

Ticagrelor monotherapy after 1–3 months of dual antiplatelet therapy (DAPT) has shown to be effective and safe after percutaneous coronary intervention (PCI), including in patients with an ST elevation myocardial infarction (STEMI). Direct omission of aspirin could further reduce bleeding complications and may reduce the incidence and expansion of intramyocardial haemorrhage (IMH), a frequent complication after revascularisation for a STEMI.

**Methods:**

This multicentre open label pilot study randomises 200 STEMI patients within 24 hours after primary PCI and before the first subsequent dose of aspirin to ticagrelor monotherapy or ticagrelor plus aspirin for twelve months. As IMH is more frequently observed after an anterior STEMI, IMH and infarct size will be determined with cardiac magnetic resonance (CMR) imaging in 60 anterior STEMI patients. In this subgroup, blood samples will be analysed for biochemical outcomes.

**Results:**

The primary safety endpoint consists of major adverse cardiac and cerebral events, and the primary efficacy endpoint is infarct size on CMR. Secondary efficacy endpoints consist of the incidence and extent of IMH determined by CMR, and of clinical bleeding events. Other endpoints include all-cause mortality and biochemical outcomes.

**Conclusion:**

The STOP-IMH pilot study compares ticagrelor monotherapy with ticagrelor plus aspirin directly after primary PCI in 200 STEMI patients. We aim to provide a signal of safety regarding ischemic events for the direct omission of aspirin after primary PCI, and to compare the infarct size by CMR between the two treatment strategies in the first week after primary PCI.

## Graphical abstract

**Figure:**
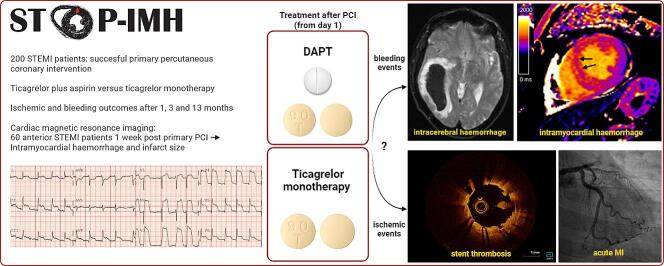

## 1 Introduction

In patients with an ST-elevation myocardial infarction (STEMI), early mechanical reperfusion of the epicardial coronary artery by primary percutaneous coronary intervention (PCI) is recommended [1]. After a PCI, treatment with dual antiplatelet therapy (DAPT), consisting of aspirin and a P2Y12 inhibitor, is recommended to reduce ischemic complications of atherosclerotic disease and stent-related ischemic outcomes [1], [2]. The advent of the more potent P2Y12 inhibitors ticagrelor and prasugrel as well as ongoing improvements in stent technology, have led to a reduction of ischemic complications [3], which questions the additional benefit of aspirin. In addition, during past decade, the non-thrombotic pharmacologic treatment of cardiovascular disease has improved significantly [4], further influencing the balance of ischemic versus bleeding risk. To prevent unnecessary overexposure to antiplatelet therapy to reduce bleeding complications, it is pivotal to re-evaluate the post-PCI antiplatelet strategy over time. A novel strategy to reduce bleeding complications is the cessation of aspirin after 1 to 3 months with continuation of the P2Y12 inhibitor, which has been demonstrated to be safe regarding ischemic events and has showed a strong reduction in major bleeding events [5], [6], [7], [8], [9], [10], [11], [12]. These findings were confirmed in subgroup analyses of STEMI patient [13], [14].

As the majority of bleeding complications in the STEMI population occur during the first month [15], a cessation of aspirin directly after primary PCI, the aspirin-free strategy, could further reduce bleeding complications.

In addition to a reduction in clinical bleeding outcomes, ticagrelor monotherapy, compared to DAPT, may also limit the cardiac damage caused by reperfusion injury. Whereas ischemia causes limited damage to the coronary endothelium in the first hours after coronary arterial occlusion, reperfusion causes immediate endothelial cell injury, which can ultimately lead to microvascular injury (MVI). In the most extensive form, MVI can result in capillary destruction and, subsequently, intramyocardial haemorrhage (IMH) [16], [17]. External compression of the microvasculature by IMH increases vascular resistance which further impairs perfusion of the distal coronary microvasculature. Further damage is caused by the cytotoxic haem to which the cardiomyocytes are then exposed. It has been demonstrated that haemorrhagic myocardial infarctions are associated with up to 80% larger infarct sizes [18], and predicts major adverse cardiac events, adverse remodelling and reduced left ventricular function [19]. Therefore, reduction of haemorrhagic damage is a potential new target. A strong association of IMH with additional GPIIb/IIIa inhibitor treatment has been demonstrated, which corroborates the concept that more aggressive antithrombotic strategies after primary PCI might aggravate IMH [20]. Therefore, the choice of antiplatelet strategy after primary PCI for STEMI may be an important target influencing the occurrence and extent of IMH.

The efficacy and safety of direct omission of aspirin after PCI is currently being investigated in patients with an acute coronary syndrome (ACS) without ST-elevation [21], but has yet to be investigated in STEMI patients. The ongoing NEOMINDSET trial [22] investigates the effect of aspirin omission within four days (or later in case of a staged non-culprit PCI) after primary PCI in all ACS patients, including STEMI patients. As opposed to direct aspirin omission after primary PCI, this strategy possibly provides more protection for ischemic events in the acute phase, without a potential benefit on the reduction of IMH. As the ischemic risk is higher after a STEMI compared to other clinical settings in which patients undergo PCI [13], [14], [23], the STOP-IMH randomised trial was designed as a pilot study to provide a signal of safety regarding the incidence of ischemic events for the direct omission of aspirin after primary PCI with the continuation of ticagrelor monotherapy for 12 months compared to ticagrelor plus aspirin for 12 months in a relatively small number of STEMI patients.

Furthermore, we aim to assess the effect of this novel antiplatelet strategy on IMH and infarct size, as determined with CMR in a subgroup of patients with an anterior STEMI.

## 2 Methods

### 2.1 Design

**Fig. 1:**
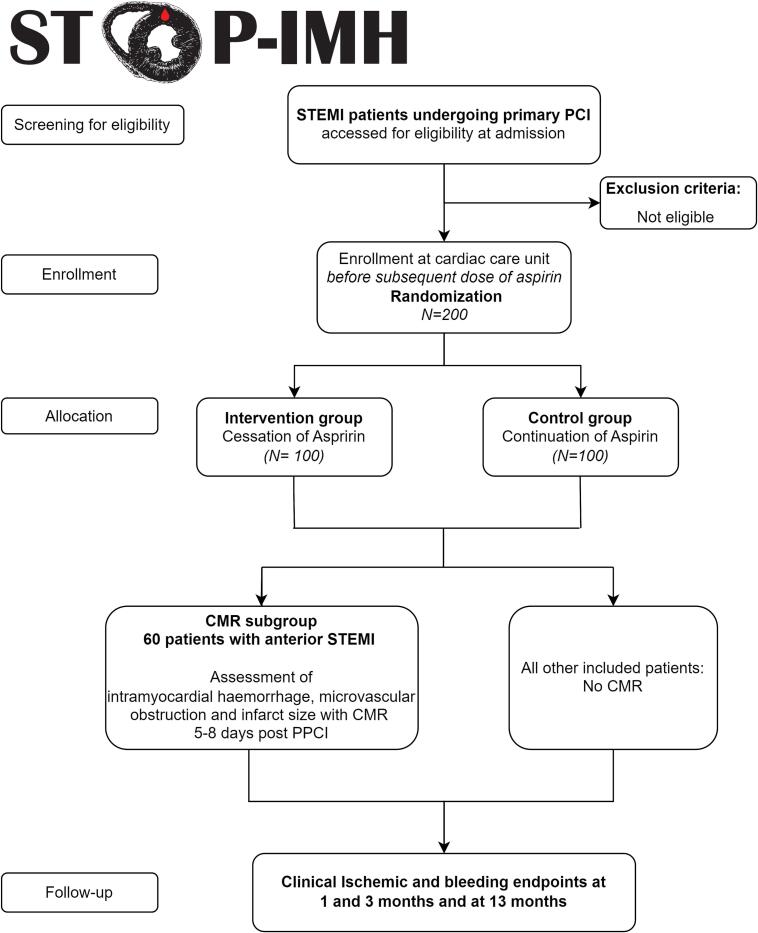
Flowchart of the study design.

The STOP-IMH pilot study is an open-label, prospective multicentre randomised clinical trial (RCT) in STEMI patients undergoing primary PCI, performed in the Netherlands. Fig. 1 presents a flow chart of the study design. Due to the pilot design, no formal power calculation has been performed. A total of 200 patients will be included and randomised within 24 h after the primary PCI and before the first dose of aspirin after primary PCI in a 1:1 ratio to ticagrelor monotherapy or ticagrelor plus aspirin for twelve months. In 60 patients with an anterior STEMI (due to a culprit in the left main (LM) artery or in the proximal- or mid left anterior descending (LAD) artery), CMR will be performed within 5 to 8 days post primary PCI. Randomisation is stratified for participation in the CMR subgroup and for participating centres.

The Medical Ethics Committee reviewed and approved this study, and the study protocol conforms to the ethical guidelines of the 1975 Declaration of Helsinki [24]. This study is registered at ClinicalTrials.gov (NCT05986968).

### 2.2 Study population

Patients aged 18 years or older are eligible for inclusion if a successful primary PCI (according to the treating physician) of the infarct-related vessel is performed with drug eluting stent placement for a clinically and electrocardiographically diagnosed STEMI. Criteria for STEMI consist of ≥0.1 mV elevation (of ≥0.2 mV in V2-V3) in two contiguous leads (in the absence of left ventricular (LV) hypertrophy or left bundle branch block (LBBB)) and reciprocal depressions. Patients will be excluded when they fulfil one of the following exclusion criteria: A known allergy or contraindication for aspirin, ticagrelor or prasugrel; a previous PCI or MI less than 12 months ago; previous cardiac surgery; pregnancy and breast feeding; concurrent use of oral anticoagulants; periprocedural use of GPIIb/IIIa inhibitors; planned surgical intervention within 12 months of PCI; creatinine clearance <30 mL/min or dialysis; PCI of stent thrombosis; suboptimal PCI result as judged by the treating interventional cardiologist; life expectancy shorter than 13 months.

### 2.3 Treatment

As inclusion and randomisation takes place after the primary PCI, all patients receive loading dose aspirin and ticagrelor before primary PCI, as per local protocol. In the intervention arm, aspirin is discontinued directly after randomization and ticagrelor monotherapy (90 mg two times daily) is continued for 12 months. In specific cases, aspirin can be reinstated before 12 months after randomisation at the discretion of the treating physician, for example after a new ischemic event, such as a new myocardial infarction or stent thrombosis. In case of a staged PCI of non-culprit lesions, an additional loading dose of aspirin is administered prior to the staged PCI, after which the aspirin is discontinued.

In the control arm, patients will receive aspirin 75–100 mg once daily and ticagrelor 90 mg twice daily for 12 months. For several reasons, DAPT can be temporarily stopped and reinstated in the control arm at the treating physician’s discretion, such as surgery, an invasive procedure, or a clinically relevant bleeding. After 12 months, antiplatelet therapy in all patients is according to the treating physician.

If patients require treatment with an oral anticoagulant during the follow-up period, for example, in case of new-onset atrial fibrillation, ticagrelor will be switched to clopidogrel, and in the control arm, aspirin will be stopped. Other (non-coagulant) co-medication should be prescribed as recommended by the current international guidelines.

### 2.4 CMR subgroup

**Fig. 2:**
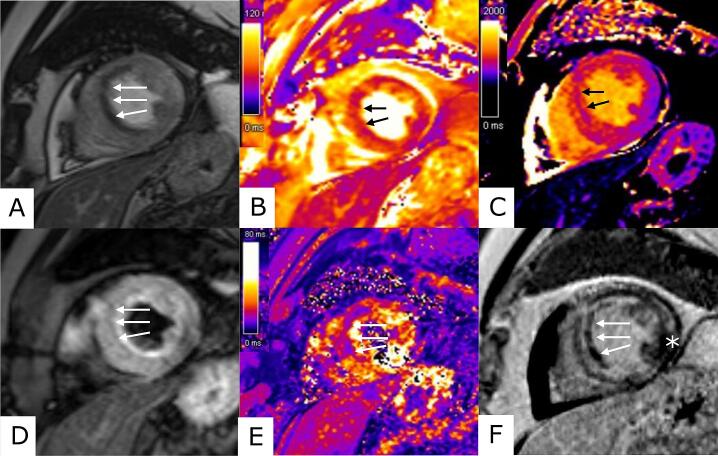
CMR images of intramyocardial haemorrhage. CMR images of a patient with an acute anteroseptal infarction with IMH. Cine showing anteroseptal akinesia, indicated by the white arrows (A). Black arrows indicate IMH in the T2 map and T1 map (B, C). White arrows indicate IMH as a hypointense core within a hyperintense area in the T2W STIR and T2* map (D, E). LGE demonstrating MVO, indicated by the white arrows. The white Asterix shows normal myocardium (F). CMR: cardiac magnetic resonance, IMH: intramyocardial haemorrhage, T2W STIR: T2-weighted short-tau inversion recovery, LGE: late gadolinium enhancement, MVO: microvascular obstruction.

Cardiac magnetic resonance (CMR) imaging provides noninvasively visualization of microvascular obstruction (MVO) (reflecting MVI) and IMH and relates this to the total infarct size and cardiac function [16], [25], [26]. Fig. 2 presents an example of IMH on CMR. Anterior infarct location is independently associated with the occurrence of IMH [20], [27] and with extensive IMH [20]. Therefore, CMR will be performed in patients with an anterior STEMI (LM or proximal or mid LAD culprit). We expect that this will be approximately 30% of the total population. Hence, 60 patients will be enrolled in the CMR subgroup. If 60 inclusions in the subgroup is reached before the total number of 200 patients have been included, the remaining anterior STEMI patients will be included as non-CMR patients. If the enrolment of subgroup patients has not yet reached 60 inclusions before the total of 200 will be enrolled, enrolment of the overall population will be stopped and only subgroup patients will be included. The timing of the CMR of 5 to 8 days after primary PCI is based on the expected expansion rate of IMH and differences in platelet aggregation between treatment groups. As both groups receive the loading dose of aspirin, no difference in platelet aggregation is expected in the first days after PCI. Thereafter, platelet inhibition will diverge between treatment groups. In the control group platelet inhibition will further increase by subsequent DAPT. In the experimental group the further inhibition of (newly formed) thrombocytes will rely on ticagrelor monotherapy. Results from earlier patient and animal studies demonstrated the greatest expansion of IMH within the first three days after primary PCI followed by a stable phase [18], [20], [28]. Decrease of IMH was demonstrated at ten days post PCI [28]. Therefore, CMR is preferably performed after several days to ensure the visibility of potential differences between treatments, but before resorption of the hematoma occurs. CMR examination will be performed on a 1.5 or 3.0-T clinical MRI scanner using a phased array cardiac receiver coil. All images are ECG-gated and acquired during mild end-expiration breath holding.

Steady-state free-precession cine imaging is used to measure LV volumes and calculate LV ejection fraction (LVEF) and to assess left ventricular segmental and global strain. T2-weighted imaging, T2 mapping and T2* mapping will be acquired in short axis planes for the detection of intramyocardial haemorrhage and infarct-related oedema. T1 maps (native and post contrast) will be acquired to quantify remote myocardial inflammation. Finally, after the administration of a gadolinium-based contrast agent (0.1 mmol/kg), LGE imaging will be performed to determine total infarct size, the transmural extent of infarction and presence and extent of microvascular obstruction.

Blood plasma samples of patients within the CMR subgroup will be stored on the day of the randomisation, the day of the CMR and during two outpatient visits (after 6 weeks and after one year) for future analyses regarding inflammatory- and coagulation response, unless logistically not feasible. Furthermore, in the CMR subgroup platelet inhibition by aspirin will be measured using VerifyNow on the day of randomisation, 2–4 days after primary PCI and on the day of the CMR, to compare the decrease of platelet inhibition by aspirin within the first week between patients receiving only the loading dose and patients in which the aspirin is continued. The VerifyNow aspirin assay measures platelet aggregation using fibrinogen and arachidonic acid. When platelets are not inhibited by aspirin, they bind to the fibrinogen on activation by the arachidonic acid. This results in agglutination of the fibrinogen, which is measured in Aspirin Reaction Units (ARU).

### 2.5 Follow-up and outcomes

Our primary safety endpoint is the composite of major adverse cardiac and cerebral events (MACCE) consisting of myocardial infarction, stent thrombosis, ischemic stroke and cardiovascular mortality.

The primary efficacy endpoint is the infarct size, determined by CMR on days 5 to 8 after primary PCI.

Secondary efficacy endpoints consist of the incidence and extent of IMH determined by CMR, and of clinical bleeding events. Bleeding events are defined according to the Bleeding Academic Research Consortium (BARC) criteria. BARC type 2 or higher bleeding events are collected. Other endpoints include all-cause mortality, echocardiographic left ventricular function between 6 and 13-months follow-up, and biochemical outcomes such as platelet inhibition to aspirin, inflammatory response and coagulation markers. Complications and adherence to medication during follow-up is retrieved by telephonic follow-up consults at one, three and thirteen months post primary PCI.

### 2.6 Data collection

Data are collected in a pseudonymised manner in Castor electronic data capture (EDC) [29]. Medical history, baseline characteristics, procedural characteristics and medication at discharge will be retrieved from the patient’s electronical health record (EHR). Complications and medication changes reported by the patients during telephonic follow-up visits will be verified in the EHR and registered in the electronic Clinical Research Form (eCRF) of Castor EDC. Changes in the antiplatelet regimen reported by the patient will be verified in the EHR, and the details about the date, duration and reason for temporary or permanent interruption or reinstatement of aspirin will be documented in the eCRF. Diagnostic imaging and biochemical results are also registered in Castor EDC.

### 2.7 Data protection and quality control

Two times per year, external monitoring will take place at each site to ensure completeness and correctness of data in the eCRF. Before the data analysis, a clinical event committee (CEC) will evaluate all endpoints to ensure the correctness of the registered complications. Each site creates and stores an identification log which is securely stored at the specific site to identify the patient to the Castor identification number if required. All study data will be stored for 25 years after the last follow-up visit.

### 2.8 Safety of study participants

A data safety monitoring board (DSMB) was installed to ensure safety of the study participants. The DSMB will periodically review the study’s safety outcomes. If three stent thromboses have occurred in the experimental arm, an interim safety analysis will be performed.

### 2.9 Organization

The STOP-IMH trial was initiated by the Department of Cardiology of the Radboud University Medical Centre and is led by a steering committee consisting of the principal investigator (PD), the coordinating investigator (EW) and two sub-investigators (RN and BY). The other participating sites are Medisch Spectrum Twente, Rijnstate ziekenhuis, Noordwest ziekenhuisgroep and Amsterdam University Medical Centre. All centres have a local PI, who is responsible for conducting the study at the respective site according to the study protocol and common rules of good clinical practice.

### 2.10 Statistical analysis

Statistical analyses will be conducted according to both the intention to treat and the per protocol principles. The main analysis will be based on the intention to treat principle. Continuous baseline demographic and clinical variables will be expressed as mean ± standard deviation (SD) for normally distributed continuous parameters, and as median (IQR) for those with a skewed distribution. Categorical data will be expressed as frequencies and proportions. For continuous non-normally distributed variables we will use the Mann-Whitney *U* test. For continuous normally distributed variables, we will use the unpaired *t*-test. Cox regression analysis will be performed to calculate the hazard ratio’s and concomitant confidence intervals to compare outcomes between treatment strategies. Risk differences between treatments will also be calculated. The main analyses will comprise of clinical outcomes in the first three months after primary PCI and of CMR results. In addition, we will report on clinical outcomes at thirteen months.

## 3 Discussion

The STOP-IMH trial is a multicentre open-label randomised pilot study, performed in the Netherlands, that compares ticagrelor monotherapy with ticagrelor plus aspirin directly after primary PCI in STEMI patients on ischemic outcomes and infarct size determined with CMR.

### 3.1 Strengths

To our knowledge, this study is the first randomised trial that compares the direct omission of aspirin with the continuation of ticagrelor monotherapy after primary PCI with ticagrelor plus aspirin in STEMI patients. In addition to the primary safety endpoint of MACCE, this study investigates the effect of the omission of aspirin on IMH and infarct size with CMR after 5–8 days. As IMH increases the infarct size and has a negative impact on the patient prognosis [18], [19], it is important to investigate whether the aspirin-free strategy diminishes the incidence and extent of IMH. These results would enable future powered studies that further investigate differences in IMH and infarct size and investigate long term effects on the left ventricular ejection fraction and prognosis between the two treatment strategies. In addition to the reduction in clinical bleeding events, an improvement in ejection fraction and prognosis would further support the choice for ticagrelor monotherapy over dual antiplatelet therapy.

As all patients receive a loading dose of aspirin, which irreversibly binds to the thrombocytes, it is important to investigate the decrease of thrombocyte inhibition in first week, especially with respect to the CMR results. By using VerifyNow, which was applied due to its feasibility and use in comparable trials [30], [31], valuable data regarding the antiplatelet response to aspirin in the first week is added to the study outcomes.

Another strength of the STOP-IMH trial, is the storage of blood plasma of patients with an anterior STEMI (i.e., the CMR subgroup) at several time points during the first year. Previous studies have demonstrated that ticagrelor has anti-inflammatory effects [32] as opposed to certain pro-inflammatory effects of aspirin [33]. As suppressing the pro-inflammatory phase and upregulating the anti-inflammatory response could diminish cardiac damage and therefore reduce infarct size, the effects of ticagrelor and aspirin on the inflammatory response are of interest in STEMI patients [34] and will therefore be analysed. Furthermore, coagulation plays an important role in both the acute and subacute phases of myocardial infarction, and STEMI patients show a disbalance in coagulation towards a hypercoagulable state which could increase the risk of thrombotic events such as myocardial infarctions and stent thrombosis [35]. To investigate differences in coagulation response between patients with or without aspirin next to ticagrelor, we also plan future analyses regarding coagulation response. Lastly, both academic and peripheral centres participate in the STOP-IMH trial, and complex PCI is not an exclusion criterion, which both help to provide an accurate representation of the current STEMI population and to increase the general applicability of the findings.

### 3.2 Weaknesses

The underpowering of results due to the pilot design is the main limitation of this study. If this study provides a signal of safety and efficacy, powered studies are required to prove the safety and efficacy of the aspirin-free strategy in STEMI patients before implementation in clinical practice may take place. Also, due to the small number of patients, the possibility of performing subgroup analyses will be limited.

#### 3.2.1 Strategies to decrease bleeding risk in STEMI

In the current study, ticagrelor is the used P2Y12 inhibitor and therefore results are not directly applicable to all P2Y12 inhibitors. A recent *meta*-analysis has shown that P2Y12 monotherapy does not increase ischaemic risk and reduces the risk of major bleeding, especially in patients with ACS [36]. However, the included studies have a short-term DAPT regime before interruption of aspirin, and STEMI patients are underrepresented. We note that one included study comparing clopidogrel monotherapy with DAPT showed worse ischemic outcomes with clopidogrel in ACS patients [37]. Therefore, more potent P2Y12 inhibition such as ticagrelor might be needed.

Another strategy to decrease bleeding risk in STEMI includes genotype-guided de-escalation. It has been shown that genotype guided DAPT, in which patients that show no loss of function of the CYP2C19 enzyme are de-escalated from ticagrelor to clopidogrel, is noninferior with regards to ischemic events and reduces bleeding events in STEMI patients [38].

## 4 Conclusion

The STOP-IMH pilot study compares ticagrelor monotherapy with ticagrelor plus aspirin directly after primary PCI in 200 STEMI patients. The primary focus of this study is to provide a signal of safety regarding ischemic events for the direct omission of aspirin after primary PCI with the continuation of ticagrelor monotherapy for 12 months compared to ticagrelor plus aspirin for 12 months, and to compare the infarct size by CMR between the two treatment strategies in the first week after primary PCI.

## CRediT authorship contribution statement

**E.C.I. Woelders:** Writing – original draft, Visualization, Project administration, Methodology, Investigation, Data curation, Conceptualization. **B. Yosofi:** Writing – original draft, Investigation, Data curation. **D.A.M. Peeters:** Writing – original draft, Data curation. **L.S.F. Konijnenberg:** Writing – original draft. **C. von Birgelen:** Writing – original draft, Investigation. **J.B. van Rees:** Writing – original draft, Investigation. **S.C.H. van den Oord:** Writing – original draft, Investigation. **A.A.C.M. Heestermans:** . **B.E.P.M. Claessen:** . **N. van Royen:** Writing – original draft, Investigation. **R.J.M. van Geuns:** Writing – original draft, Investigation. **R. Nijveldt:** Writing – original draft, Supervision, Methodology, Investigation, Funding acquisition, Conceptualization. **P. Damman:** Writing – original draft, Supervision, Project administration, Methodology, Funding acquisition, Data curation, Conceptualization.

## Funding

The STOP-IMH is funded by the Department of Cardiology of the Radboud University Medical Centre, Nijmegen, the Netherlands.

## Declaration of competing interest

The authors declare the following financial interests/personal relationships which may be considered as potential competing interests: Peter Damman has received research grants from Abbott, Philips, and AstraZeneca. Robin Nijveldt has received research grants from Philips Volcano and Biotronik. Robert Jan M. van Geuns has received grants and personal fees from Boston Scientific, Abbott Vascular, AstraZeneca and Amgen, and grants from InfraRedx. Bimmer Claessen has received speaker and/or consultancy fees from Abbott Vascular, Abiomed, Amgen, Boston Scientific, BBraun, and Sanofi, educational grants from Boston Scientific and Abiomed, and research grants from Philips, Novo Nordisk, BBraun and Nipro/Infraredx. Niels van Royen has received research grants from Philips, Biotronik, Abbott and Medtronic and speaker fees from Abbott, RainMed, Microport and Bayer. Clemens von Birgelen reported institutional research grants from Abbott Vascular, Biotronik, and Medtronic, outside the present study. The other authors report no relationships that could be construed as a conflict of interest.

## References

[1] Ibanez B., James S., Agewall S. (2018). 2017 ESC Guidelines for the management of acute myocardial infarction in patients presenting with ST-segment elevation: the Task Force for the management of acute myocardial infarction in patients presenting with ST-segment elevation of the European Society of Cardiology (ESC). Eur. Heart J..

[2] Valgimigli M., Bueno H., Byrne R.A. (2018). 2017 ESC focused update on dual antiplatelet therapy in coronary artery disease developed in collaboration with EACTS: the Task Force for dual antiplatelet therapy in coronary artery disease of the European Society of Cardiology (ESC) and of the European Association for Cardio-Thoracic Surgery (EACTS). Eur. Heart J..

[3] Feng W.H., Hsieh I.C., Li Y.H. (2021). P2Y12 inhibitor monotherapy after percutaneous coronary intervention: is it safe to abandon aspirin?. Acta Cardiol Sin..

[4] Baigent C., Blackwell L., Collins R. (2009). Aspirin in the primary and secondary prevention of vascular disease: collaborative meta-analysis of individual participant data from randomised trials. Lancet.

[5] Vranckx P., Valgimigli M., Jüni P. (2018). Ticagrelor plus aspirin for 1 month, followed by ticagrelor monotherapy for 23 months vs aspirin plus clopidogrel or ticagrelor for 12 months, followed by aspirin monotherapy for 12 months after implantation of a drug-eluting stent: a multicentre, open-label, randomised superiority trial. Lancet.

[6] Mehran R., Baber U., Sharma S.K. (2019). Ticagrelor with or without aspirin in high-risk patients after PCI. N. Engl. J. Med..

[7] Hahn J.Y., Song Y.B., Oh J.H. (2019). Effect of P2Y12 inhibitor monotherapy vs dual antiplatelet therapy on cardiovascular events in patients undergoing percutaneous coronary intervention: the SMART-CHOICE randomized clinical trial. J. Am. Med. Assoc..

[8] Watanabe H., Domei T., Morimoto S. (2019). Effect of 1-month dual antiplatelet therapy followed by clopidogrel vs 12-month dual antiplatelet therapy on cardiovascular and bleeding events in patients receiving PCI: the STOPDAPT-2 randomized clinical trial. J. Am. Med. Assoc..

[9] Kim B.K., Hong S.J., Cho Y.H. (2020). Effect of ticagrelor monotherapy vs ticagrelor with aspirin on major bleeding and cardiovascular events in patients with acute coronary syndrome: the TICO randomized clinical trial. J. Am. Med. Assoc..

[10] Valgimigli M., Frigoli E., Heg D. (2021). Dual antiplatelet therapy after PCI in patients at high bleeding risk. N. Engl. J. Med..

[11] O'Donoghue M.L., Murphy S.A., Sabatine M.S. (2020). The safety and efficacy of aspirin discontinuation on a background of a P2Y12 inhibitor in patients after percutaneous coronary intervention: a systematic review and meta-analysis. Circulation.

[12] Giacoppo D., Matsuda Y., Fovino L.N. (2021). Short dual antiplatelet therapy followed by P2Y12 inhibitor monotherapy vs. prolonged dual antiplatelet therapy after percutaneous coronary intervention with second-generation drug-eluting stents: a systematic review and meta-analysis of randomized clinical trials. Eur. Heart J..

[13] Gamal A.S., Hara H., Tomaniak M. (2021). 'Ticagrelor alone vs. dual antiplatelet therapy from 1 month after drug-eluting coronary stenting among patients with STEMI': a post hoc analysis of the randomized GLOBAL LEADERS trial. Eur. Heart J. Acute Cardiovasc. Care.

[14] Lee S.J., Cho J.Y., Kim B.K. (2021). Ticagrelor monotherapy versus ticagrelor with aspirin in patients with ST-segment elevation myocardial infarction. J. Am. Coll. Cardiol. Intv..

[15] Giustino G., Mehran R., Dangas G.D. (2017). Characterization of the average daily ischemic and bleeding risk after primary PCI for STEMI. journal of the. Am. Coll. Cardiol..

[16] Konijnenberg L.S.F., Damman P., Duncker D.J. (2020). Pathophysiology and diagnosis of coronary microvascular dysfunction in ST-elevation myocardial infarction. Cardiovasc. Res..

[17] Robbers L.F., Eerenberg E.S., Teunissen P.F. (2013). Magnetic resonance imaging-defined areas of microvascular obstruction after acute myocardial infarction represent microvascular destruction and haemorrhage. Eur. Heart J..

[18] Liu T., Howarth A.G., Chen Y. (2022). Intramyocardial Hemorrhage and the “Wave Front” of Reperfusion Injury Compromising Myocardial Salvage. J. Am. Coll. Cardiol..

[19] Husser O., Monmeneu J.V., Sanchis J. (2013). Cardiovascular magnetic resonance-derived intramyocardial hemorrhage after STEMI: influence on long-term prognosis, adverse left ventricular remodeling and relationship with microvascular obstruction. Int. J. Cardiol..

[20] Amier R.P., Tijssen R.Y.G., Teunissen P.F.A. (2017). Predictors of intramyocardial hemorrhage after reperfused ST-segment elevation myocardial infarction. J. Am. Heart Assoc..

[21] van der Sangen N.M.R., Kucuk I.T., Sivanesan S. (2023). Less bleeding by omitting aspirin in non-ST-segment elevation acute coronary syndrome patients: rationale and design of the LEGACY study. Am. Heart J..

[22] Guimaraes P.O., Franken M., Tavares C.A.M. (2023). P2Y12 inhibitor monotherapy versus dual antiplatelet therapy in patients with acute coronary syndromes undergoing coronary stenting: rationale and design of the NEOMINDSET Trial. EuroIntervention.

[23] Neumann F.J., Sousa-Uva M., Ahlsson A. (2019). 2018 ESC/EACTS Guidelines on myocardial revascularization. Eur. Heart J..

[24] World Medical Association Declaration of Helsinki: ethical principles for medical research involving human subjects. JAMA 284 (23) (2000) 3043–3045. 11122593

[25] Nijveldt R., Beek A.M., Hirsch A. (2008). Functional recovery after acute myocardial infarction: comparison between angiography, electrocardiography, and cardiovascular magnetic resonance measures of microvascular injury. J. Am. Coll. Cardiol..

[26] van Kranenburg M., Magro M., Thiele H. (2014). Prognostic value of microvascular obstruction and infarct size, as measured by CMR in STEMI patients. J. Am. Coll. Cardiol. Img..

[27] Reinstadler S.J., Stiermaier T., Reindl M. (2019). Intramyocardial haemorrhage and prognosis after ST-elevation myocardial infarction. Eur. Heart J. Cardiovasc. Imaging.

[28] Carrick D., Haig C., Ahmed N. (2016). Temporal evolution of myocardial hemorrhage and edema in patients after acute ST-segment elevation myocardial infarction: pathophysiological insights and clinical implications. J. Am. Heart Assoc..

[29] Castor EDC, Castor Electronic Data Capture, 2019.

[30] van der Sangen N.M.R., Claessen B., Kucuk I.T. (2023). Single antiplatelet therapy directly after percutaneous coronary intervention in non-ST-segment elevation acute coronary syndrome patients: the OPTICA study. EuroIntervention.

[31] van Leeuwen M.A.H., van der Hoeven N.W., Janssens G.N. (2019). Evaluation of microvascular injury in revascularized patients with ST-segment-elevation myocardial infarction treated with ticagrelor versus prasugrel. Circulation.

[32] Thomas M.R., Outteridge S.N., Ecob R. (2014). Ticagrelor inhibits release of pro-inflammatory cytokines TNF and IL-6 during human endotoxaemia. Eur. Heart J..

[33] Kiers D., van der Heijden W.A., van Ede L. (2017). A randomised trial on the effect of anti-platelet therapy on the systemic inflammatory response in human endotoxaemia. Thromb. Haemost..

[34] Ong S.B., Hernandez-Resendiz S., Crespo-Avilan G.E. (2018). Inflammation following acute myocardial infarction: multiple players, dynamic roles, and novel therapeutic opportunities. Pharmacol. Ther..

[35] Teunissen P.F.A., Tijssen R., van Montfoort M.L. (2016). Kinetics of coagulation in ST-elevation myocardial infarction following successful primary percutaneous coronary intervention. Thromb. Res..

[36] Valgimigli M., Hong S.J., Gragnano F. (2024). De-escalation to ticagrelor monotherapy versus 12 months of dual antiplatelet therapy in patients with and without acute coronary syndromes: a systematic review and individual patient-level meta-analysis of randomised trials. Lancet.

[37] Watanabe H., Morimoto T., Natsuaki M. (2022). Comparison of clopidogrel monotherapy after 1 to 2 months of dual antiplatelet therapy with 12 months of dual antiplatelet therapy in patients with acute coronary syndrome the STOPDAPT-2 ACS randomized clinical trial. JAMA Cardiol..

[38] Claassens D.M.F., Vos G.J.A., Bergmeijer T.O. (2019). A genotype-guided strategy for oral P2Y(12) inhibitors in primary PCI. N. Engl. J. Med..

